# Aligned membranes regulate wound healing via MMP12 secreted by macrophages

**DOI:** 10.1371/journal.pone.0317194

**Published:** 2025-01-15

**Authors:** Hongli Ma, Limin Gong, Xi Yang, Hui Fang, Jiacai He, Chenbing Wang

**Affiliations:** College & Hospital of Stomatology, Key Laboratory of Oral Diseases Research of Anhui Province, Anhui Medical University, Hefei, China; Instituto do Cancer do Estado de Sao Paulo / University of Sao Paulo, BRAZIL

## Abstract

Aligned electrospinning membranes (Align) have demonstrated the potential to enhance wound healing by establishing a regenerative microenvironment surrounding the wound; However, the precise mechanism underlying its facilitation of healing remains unclear. To elucidate aligned electrospun fiber membrane’s role in accelerating wound healing and improving its quality, we conducted a comprehensive analysis. Firstly, in vivo experiments confirmed that Align promotes wound healing. Through combined bulk RNA sequencing and single-cell RNA sequencing, we identified that MMP12^+^ macrophages exhibit high expression of MMP12 during the early stage of wound remodeling, thereby inhibiting fibroblast migration and reducing scar formation after wound closure. Finally, both in vitro and in vivo experiments further validated the role of MMP12 in promoting wound healing and suppressing fibroblast migration. Our findings reveal that Align effectively enhances skin wound healing by upregulating MMP12 expression while inhibiting fibroblast migration. These insights provide valuable knowledge on how Align promotes efficient scar-free wound healing and serve as a theoretical foundation for developing more effective biological dressings.

## 1.Introduction

Aligned membranes exhibit a diverse array of applications within the domain of skin tissue engineering [1–3]. Due to their ability to deliver a range of biochemical and physical signals, they play a crucial role in regulating cellular behaviors and modulating the immune response [4,5]. For instance, studies have reported that fibroblasts can migrate over long distances in a highly coordinated manner at a constant speed on aligned membranes [6]. The cells that are attached to the radially aligned nanofibers can migrate more efficiently from the surrounding healthy tissue to the central injured site, thereby accelerating the closure of the wound [7]. Critically, aligned membranes have the potential to enhance the normal differentiation and outgrowth of vascular smooth muscle cells, thereby serving as a valuable tool in the construction of blood vessels for tissue engineering applications [1,8]. An important application of aligned membranes is to minimize the formation of scar tissue in skin wounds [9]. As the primary defense against external environmental factors, skin wound healing is a highly dynamic and meticulously coordinated process. It necessitates complex interactions among various resident cell types to sustain barrier function [10,11]. The classical wound healing process in adult skin is traditionally categorized into four sequential phases that temporally and spatially overlap: a hemostasis preparation phase (0 to several hours post-wounding), a pro-inflammatory initiation phase (1 to 3 days), a wound restoration phase (4 to 21 days), and a resolution and remodeling phase (21 days to over 1 year). The duration of the latter phase is primarily influenced by the severity of the wound [12]. The disruption of any of these phases significantly impairs the healing process, resulting in conditions such as chronic non-healing ulcers or excessive scarring. The normal physiological functions are adversely affected, leading to limitations in tissue movement and consequently causing negative psychological impacts on patients [13,14], imposing a significant burden on individuals and society, both in terms of health and economic factors [15–17]. To date, several targeted treatments have been developed, primarily involving the local application of healing factors. However, these approaches exhibit significantly limited clinical efficacy due to constraints in their delivery methods and duration of action [18]. Therefore, there is an urgent need to identify novel therapeutic targets and to redevelop more effective treatment strategies.

Most prior research on minimizing scar formation in wounds has concentrated on various cellular components of the healing process, including reparative cells, growth factors, and the biomechanical environment [19–24]. The effects of aligned membranes on fibroblasts, the primary cell type involved in scar formation [25], have not been thoroughly investigated. Previous studies have shown that the proper functioning of matrix metalloproteinases (MMPs) is essential for the migration of fibroblasts on the extracellular matrix (ECM) [26,27], and effectively modulates the formation of scar tissue [28]. The activity of matrix metalloproteinases (MMPs) is modulated by their inhibitors, known as tissue inhibitors of metalloproteinases (TIMPs) [29]. MMP12 is a matrix metalloproteinase secreted by macrophages, also known as matrix metallopeptidase 12 or macrophage elastase. It is a member of the matrix metalloproteinase (MMP) family and plays a crucial role in regulating the degradation of the extracellular matrix [30,31], It has been reported that it actively regulates cell migration and provides protection against corneal fibrosis during the process of corneal wound healing [32,33]. Another study has revealed that MMP12 inhibits inflammation and neovascularization after corneal injury by regulating CCL2 [34].

Recently, we have demonstrated that aligned membranes offer significant advantages in the repair of soft tissues and can actively modulate the immune response [35], and speculated that MMP12 plays a significant role in the regulation of scar formation. While aligned membranes are known to be critical during the wound healing process, the effects and implications of these membranes on minimizing scar formation within this context have not been thoroughly investigated. The present study aims to address this clinically relevant issue and propose potential treatment strategies for reducing scar formation and enhancing wound healing under conditions involving aligned membranes.

## 2. Materials and methods

### 2.1. Preparation of aligned membranes via electrospinning

To fabricate the aligned membranes, PLGA (Polylactic acid glycolic acid, PLGA) (20% w/v) and FC (Fish collagen, FC) (2% w/v) were dissolved in HFIP (Hexafluoroisopropanol, HFIP) in a shaker of 240 rpm at 25°C.

Subsequently, the prepared solutions were utilized to fabricate aligned fibrous membranes. The solutions were loaded into a plastic syringe equipped with a flat-tipped 21 G stainless steel needle. A high voltage of -2/5 kV and a distance of 11 cm were established between the needle and the roller collector, which was set at 2800 rpm and covered with aluminum foil. The solution was delivered at a constant rate of 0.018 mm/min, regulated by a precision syringe pump.

The resulting aligned membranes were dried in a vacuum oven at 25°C until complete volatilization of the solvent occurred. The fiber diameter measured 319 ± 100 nm (A300), after which the membranes were cut into circular shapes (8 mm) and sterilized using γ-irradiation prior to animal implantation experiments.

### 2.2. Animal experiments

#### 2.2.1 Ethical approval

All procedures were approved by Laboratory Animal Ethics Committee of Anhui Medical University (NO.LLSC20231122).

#### 2.2.2 Full-thickness skin wound model and implantation procedures

The experimental animals (C57BL/6J mice aged 8 weeks with an average weight of 20 g, Male) obtained from the experimental animal center of Anhui Medical University had ad libitum access to both feed and tap water. The healthy status of the mice was checked every day during the experimental period. After conventional fasting for 12 h, with the instructions of the Animal House, the experimental animals were intraperitoneally anaesthetized with Sodium pentobarbital (1%, 0.03 ml/10g body weight) after Inhalation of isoflurane (In order to alleviate the fear of mice). Subsequently, a circular defect with a diameter of 6 mm was surgically created on the middle skin of the dorsum following appropriate skin preparation and disinfection using 70% alcohol. After transplanting aligned membranes (with uniform specifications for small discs having a diameter of 8 mm) in the Align group, saline was added in the control group during the initial phase of the animal experiments. To replicate the wound healing process observed in humans, a mice-splinted model was employed to reduce wound contraction caused by the panniculus carnosus in rodents [36], the skin wounds were covered with 3M™Tegaderm™ and fixed on the latex ring with 5–0 silk suture. After the operation, the body temperature of the mice was restored using a heating lamp and an insulation blanket until the mice woke up. The experimental animals received routine postoperative nursing [37]. In the second part of animal experiment, the experimental mice were divided into four groups with randomness: mice wounds transplanted with the saline (Ctr group), aligned membranes (Align group); Aligned membranes plus MMP12 (MMP12 group) and aligned membranes plus MMP408 (MMP408 group). The animals were euthanatized on 7 and 14 POD With an overdose of sodium pentobarbital (1%, 0.1ml/10g), after surgery to harvest the tissue samples for general analysis.

#### 2.2.3 Single cell RNA sequencing and transcriptome sequencing

Of these, three replicates of skin wounds from mice in the Control (Ctr) and Alignment (Align) groups were analyzed for transcriptomic assessment. At POD 7, a tissue sample was excised approximately 5 mm outside the periboundary of the wound using ophthalmic scissors and tissue forceps. Three independent replicates were prepared for both the Align group and the Control group, which were subsequently stored in liquid nitrogen. The transcriptome sequencing analysis was commissioned to Novogene Bioinformatics Technology Co. Ltd. For the scRNA-seq experiments, experimental mice were randomly assigned to two groups: the Align group (Align, n = 5) and the Control group (Ctr, n = 6) on 14 POD. Following the manufacturer’s protocols, tissues were washed with pre-chilled PBS buffer and preserved in tissue preservation solution, and sequencing were performed at the BD Rhapsody of NovelBio (China).

#### 2.2.4 Histopathology, immunohistochemistry and immunofluore-scence microscopy

Wound tissue consisted of full-thickness sections obtained from the skin wounds for routine histological examination. The samples were preserved in 4% paraformaldehyde for a minimum of 48 hours prior to undergoing dehydration with ethanol and xylene. Hematoxylin and eosin (H&E) staining, along with Masson’s trichrome staining was conducted to facilitate the observation of re-epithelialization and collagen fiber deposition. For the evaluation of hair follicle formation and wound fibrosis, immunohistochemical staining was conducted for Lgr5+ (DF2816, Affinity, 1:100) and α-SMA (AF1032, Affinity, 1:100). Additionally, immunofluorescence staining was performed to assess keratinization and the presence of new hair follicles using cytokeratin 5 (K5, ab52635, Abcam, 1:200) and cytokeratin 10 (K10, ab76318, Abcam, 1:150), as well as for MMP12 (sc-390863, 1:200).

### 2.3. Invitro cell culture experiments

#### 2.3.1 Cell culture

The fibroblasts (L929) and macrophages (RAW264.7) were obtained from West China School of Stomatology, Sichuan University. The L929 and RAW264.7 were cultured in high glucose medium (DMEN; Gibco, USA) supplemented with 10% fetal bovine serum (FBS, Gibco, USA) and a 1% mixture of penicillin/streptomycin (MP Biomedicals, USA) at 37°C with 5% CO2 atmosphere. The culture medium was replaced every other day in the L929 and RAW264.7 culture cycle. These aligned membranes were preprocessed by immersing in a 75% ethanol solution for 0.5h. Subsequently, the prepared membranes were sterilized by γ-irradiation for cell experiment. The RAW264.7 cells (5 × 10^4 cells/well) were seeded in a 24-well plate with an Aligned electrospun membrane featuring fibers of 300 nm in diameter at the bottom. The cells were cultured for two days in a cell incubator maintained at 37°C and 5% CO2, after which the supernatant of the medium was collected; Under the same conditions, the culture medium without RAW264.7 was also collected for cell experiment.

#### 2.3.2 Cell migration

The L929 (1×105 cells/well) were seeded in the 24- plate at 37°C with 5% CO2 atmosphere. After scratch with 200 μl pipette tip, different stimuli. In the first cell migration experiment were divided into two groups: Align Group was added conditioned medium derived from RAW264.7 on aligned membrane with a fiber diameter of 300 nm (diluted at 1:3); Ctr Group was added conditioned medium containing only A300 without macrophages (also diluted at 1:3).The second cell migration experiment was divided into four groups: the Control group (Ctr, Added PBS), the MMP12 group(MMP12, 20 ng/ml, Biolegend, U.S.A), the MMP408 group(the inhibitor of MMP12, 20 ng/ml, Invivogen, U.S.A), and the MMP12 + MMP408 group. At the given time points (2d), the L929 in the plate were visualized with the inverted microscope (Leica, Germany) for evaluating the migration of cells. The staining of IF was performed for evaluating the expression of MMP12 (sc-390863, 1:200) and observing the cell morphology (FITC-labelled Phalloidin, Solarbio, U.S.A).

### 2.4. Quantification and statistical analysis

Data are expressed as the mean±standard error (S.e.m). Statistical analysis was performed with the Student’ s t-test and the Tukey post hoc test by analysis of variance under the condition of normal distribution, Using Case Viewer, Origin 2019 and Graphpad Prism 8.0 software. A value of p < 0.05 was considered statistically significant (*p < 0.05, **p < 0.01, ***p < 0.001). At least three independent assays were conducted to obtain repeatable data if not distinctively explained.

## 3. Results

### 3.1. Align scaffold membranes reduced wound scarring and promoted wound healing

**Fig 1:**
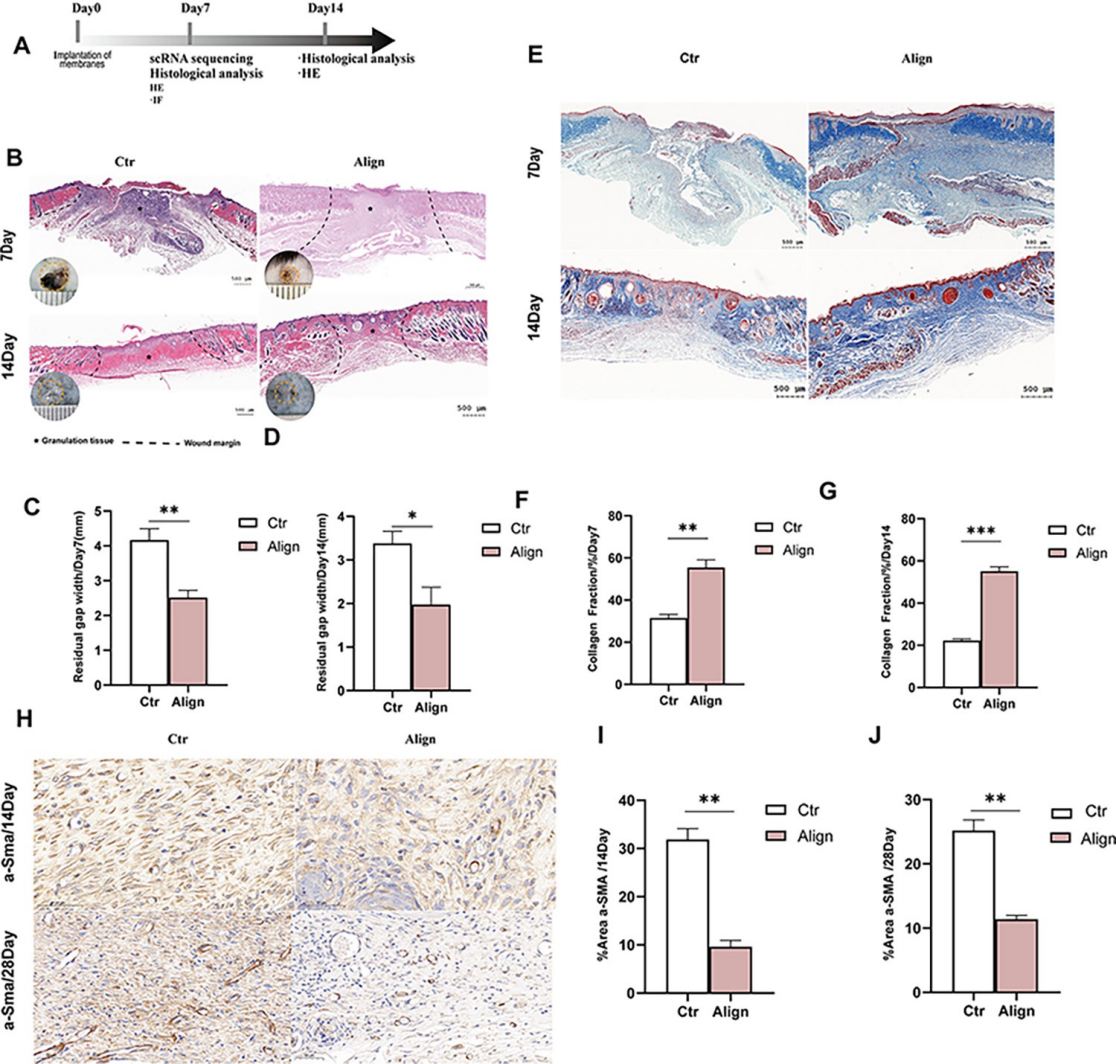
Effect of Align membrane on promoting skin wound healing in mice. (A) Workflow for evaluating the wound healing outcomes; (B) Residual gap of H&E of skin defects and at POD 7and14; (C, D) corresponding analysis; (E) The histological results for Masson staining at POD 7and 14; (F, G) corresponding analysis; (H) IHC results for a-SMA of wounds at POD14 and 28 in Control group and Align group; (I) corresponding analysis. (n = 3 for each group) Data and error bars represent the mean±s.e.m. ***P<0.001, **P<0.01, *P<0.05 and ns/no significance. Unpaired t test Student’s t-test for data in C, D, F, G, I and J.

The workflow for evaluating the potential of aligned membranes on skin wound healing was summarized in Fig 1A. First, the mice were divided into two groups: the Align group and the Control group, followed by full-thickness wound treatment (6 mm in diameter). Subsequently, we placed the Align scaffold membrane onto the wounds of each mouse in the Align group, while the Control group received a saline solution. In clinical applications, wound dressings are anticipated to enhance the healing process. Consequently, this study evaluated the soft tissue repair capabilities of A300 utilizing a mouse skin defect model, as illustrated in Fig 1B and 1C. In cases of mouse skin defects at 7 days post-operation (POD) and at 14 POD, the remaining wound area implanted with the aligned electrospun membrane in the Align group was significantly smaller than that of the Ctrl group at all time points assessed. Overall findings indicate that the A300 group indeed facilitated more effective repair of skin defects in mice. Subsequently, Masson’s trichrome staining was employed to evaluate the local deposition of collagen fiber and the induction of dermal repair in the Align On POD 7,the collagen fibers of the control group were irregularly arranged in the granulation tissue, while in the Align group, the pattern was more layered and organized; On POD 14, due to the activation of fibroblast and wound remodeling, deposited collagen was future mature in Control, and more abutment, similar to the normal dermis structure in Align (Fig 1E). On POD 7 and 14, The collagen deposition ratio in the Align group was significantly higher than that in the Ctr group (Fig 1F and 1G). Additionally, the activation of myofibroblasts is essential for scar deposition [38], Alpha-smooth muscle actin (α-SMA) is a marker of fibroblast activation and myofibroblast formation, its increased expression is a mark of fibrosis, and scarring potential is assessed by a-SMA immunohistochemistry (IHC) staining to assess the degree of fibrosis in the wound at POD 14,28 (Fig 1H), the expression level of αSMA in the Align group was significantly lower than that in the Ctr group (Fig 1I and 1J), indicating that the quantity of myofibroblasts in the Align group is less than that in the Ctr group.

**Fig 2:**
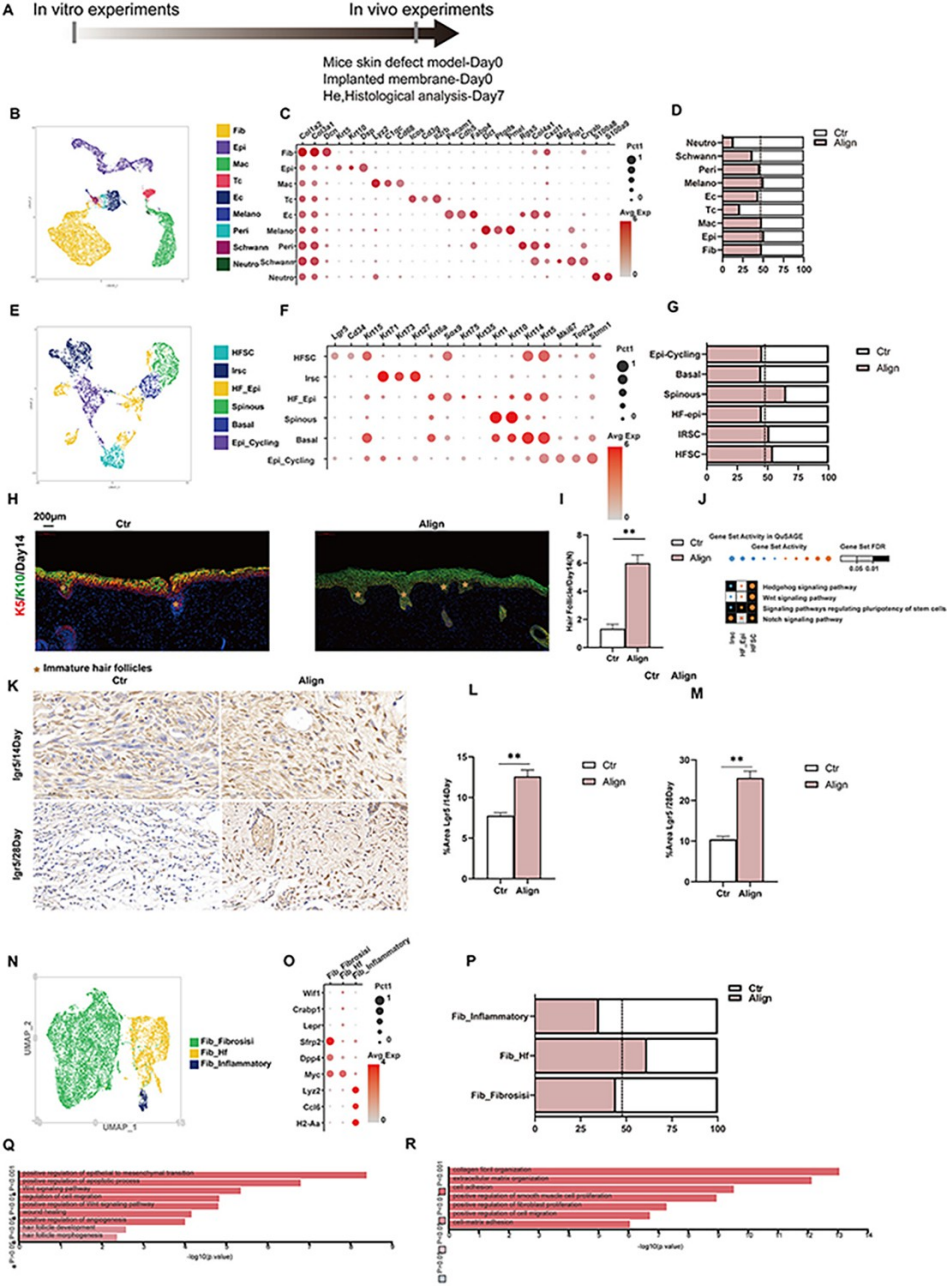
Further evaluate the effect of membrane alignment on wound healing in mice by scRNA-seq at POD 14. (A) Workflow for evaluating the wound healing outcomes by scRNA-seq at POD 14; (B) The UMAP reduction results reveals 9 different groups in wound; (C) The marker genes are listed on the right of the dot plot.; (D) The proportion of each cell type of the whole wounds in Align and Ctr; (E) The UMAP reduction results for the subclusters of epidermal cells; (F) The marker genes are listed on the right of the dot plot; (G) The proportion of subclusters of epidermal cells; (H) K5(red)/K10(green)-positive images of IF staining of skin defects at POD14; (I) Semi-quantitative Analysis of Hair Follicle Count in Wounds; (J) The heatmap of Gene Set Activity and Gene Set FDR in QuSAGE in Align and Control; (K) the IHC results for Lgr5 of wounds at POD 14 and 28 in Control group and Align group; (L,M)corresponding analysis; (N) The UMAP reduction results for the subclusters of Fibroblast; (O) The marker genes are listed on the right of the dot plot; (P) The proportion of subclusters of Fibroblast; (Q) The proportion and Gene Ontology (GO) enrichment results of subclusters of the Fibroblast of Fib_Hf; (R) Fib_Fibrosisi in Control group and Align group. Data and error bars represent the mean±s.e.m. **P<0.01, *P<0.05 and ns/no significance by Unpaired t test Student’s t-test for data in I, L and M.

To further investigate the mechanism by which Align membranes facilitate skin wound healing, we conducted single-cell RNA sequencing (scRNA-seq) on post-operative day 14, utilizing control samples (n = 6) and aligned samples (n = 5). The workflow is summarized in Fig 2A.This time point corresponds with the completion of wound re-epithelialization and a robust expression of α-SMA [39]. After quality control, 23 distinct clusters encompassing 9 cell types were identified via UMAP reduction of Seurat (Fig 2B). We annotated these cell types by known genes(Fig 2C and 2D): Fbi (*Col1a2*^*+*^, *Col3a1*^*+*^, *Dcn*^*+*^*)*, Epi (*Krt5*^*+*^, *Krt10*^*+*^, *Lgr5*^*+*^, *Dsp*^*+*^*)*, Monocyte-derived macrophages /Mono_DC (*Lyz2*^*+*^,*CD74*^*+*^*)*, T cells /TC *(Cd3d*^*+*^, *Trac*^*+*^, *Trdc*^*+*^*)*,Endothelial cells /EC *(Pecam1*^*+*^, *Cldn5*^*+*^*)*,Melanocytes /Melano*(Dct*^*+*^, *Ptgds*^*+*^*)*,Pericytes /Peri *(Rgs5*^*+*^, *Sparcl1*^*+*^*)*,Schwann cells / Schwann *(Mpz*^*+*^, *Plp1*^*+*^*)*, Neutrophils /Neutro *(S100a8*^*+*^, *Retnlg*^*+*^*)*.

Subsequently, considering the outcomes of accelerated wound closure and reduced fibrosis observed in the Align group, We studied subclusters of epidermal cells(Fig 2E) and fibroblasts(Fig 2N), annotated these cell types by known genes(Fig 2F and 2G): HFSCs (*Cd34*^*+*^, *lgr5*^*+*^, *krt17*^*+*^*)*, Irsc(*Krt71*^*+*^, *Krt73*^*+*^, *Krt27*^*+*^*)*, Hf_Epi*(Krt6a*^*+*^,*Sox9*^*+*^,*Krt75*^*+*^*)*, Spinous Cell*(Krt1*^*+*^,*krt10*^*+*^*)*, Bassal*(Krt5*^*+*^, *Krt15*^*+*^*)*, Epi_Cycling *(Mki67*^*+*^,*Top2a*^*+*^,*Stmn*^*+*^*)*; Fibroblasts are divided into the following three subclusters(Fig 2N and 2O): Fib_Fibrosisi *(Sfrp2*^*+*^, *Dpp4*^*+*^, *Myc*^*+*^*)* [40], associated with hair follicle formation/Fib_Hf*(Wif1*^*+*^, *Crabp1*, *Lepr*^*+*^*)*, Fib_Inflammatory *(Lyz2*^*+*^, *Ccl6*^*+*^, *H2-Aa*^*+*^*)*. To facilitate the generation of additional cells for wound coverage, epidermal stem cells located in the interfollicular epidermis at the wound edges, as well as those found in adjacent hair follicle bulges and sebaceous glands, begin to proliferate approximately 2 to 3 days post-injury [41–43]. Then we observed that the abundance of Krt10+ mature epidermal cells was significantly upregulated in comparison to the control group, which facilitated the establishment of the epithelial barrier (S1C Fig). The fluorescent immunostaining of Krt5+/Krt10+ was subsequently performed to assess the re-epithelization of the wound (Fig 2H and 2I). The scRNA-seq results indicated a slight increase in the expression and abundance of Krt10/Krt1 intensity (S1D and S1E Fig). It has been reported that hair follicles (HFs) are closely related to skin tensile strength and are one of the important indicators of skin functional healing [44]. We observed that the abundance of hair follicle stem cells *(HFSCs*: *Lgr5+*, *CD34+*) in the Align group was significantly higher than that in the Control group (Fig 2G). Lgr5+ (Leucine-rich repeat-containing G protein-coupled receptor 5) hair follicle stem cells exhibit the most pronounced and regular changes throughout the hair follicle cycles, as shown in (S1A Fig), which is essential for hair regeneration and wound healing [45,46]. Subsequently, Lgr5+ immunohistochemical staining demonstrated enhanced hair follicle regeneration in the Align group (Fig 2K and 2L). Additionally, the expression intensity of Lgr5 showed a slight increase in the scRNA-seq results (S1B Fig). Meanwhile, in the subcluster of fibroblasts, it is evident that the Fib_Hf ratio has significantly increased (Fig 2P), while the Fib_Fibrosis ratio has decreased. The corresponding genes have been upregulated (S1F and S1G Fig) and are involved in hair follicle development, wound healing, and fibrosis, respectively, as indicated by KEGG.GO analysis through scRNA-seq (Fig 2Q and 2R). It was confirmed that Align exhibited a greater potential for follicle regeneration and a higher level of re-epithelialization [47] compared to Control, which played a crucial role in facilitating wound closure and promoting scarless healing.

In summary, the aligned ECM scaffold membrane significantly enhances the regeneration of epidermal function and facilitates dermal repair processes. This results in accelerated wound healing and a more abundant and organized deposition of collagen.

### 3.2. The expression of MMP12 in Align increased and inhibited the migration ability of fibroblasts

**Fig 3:**
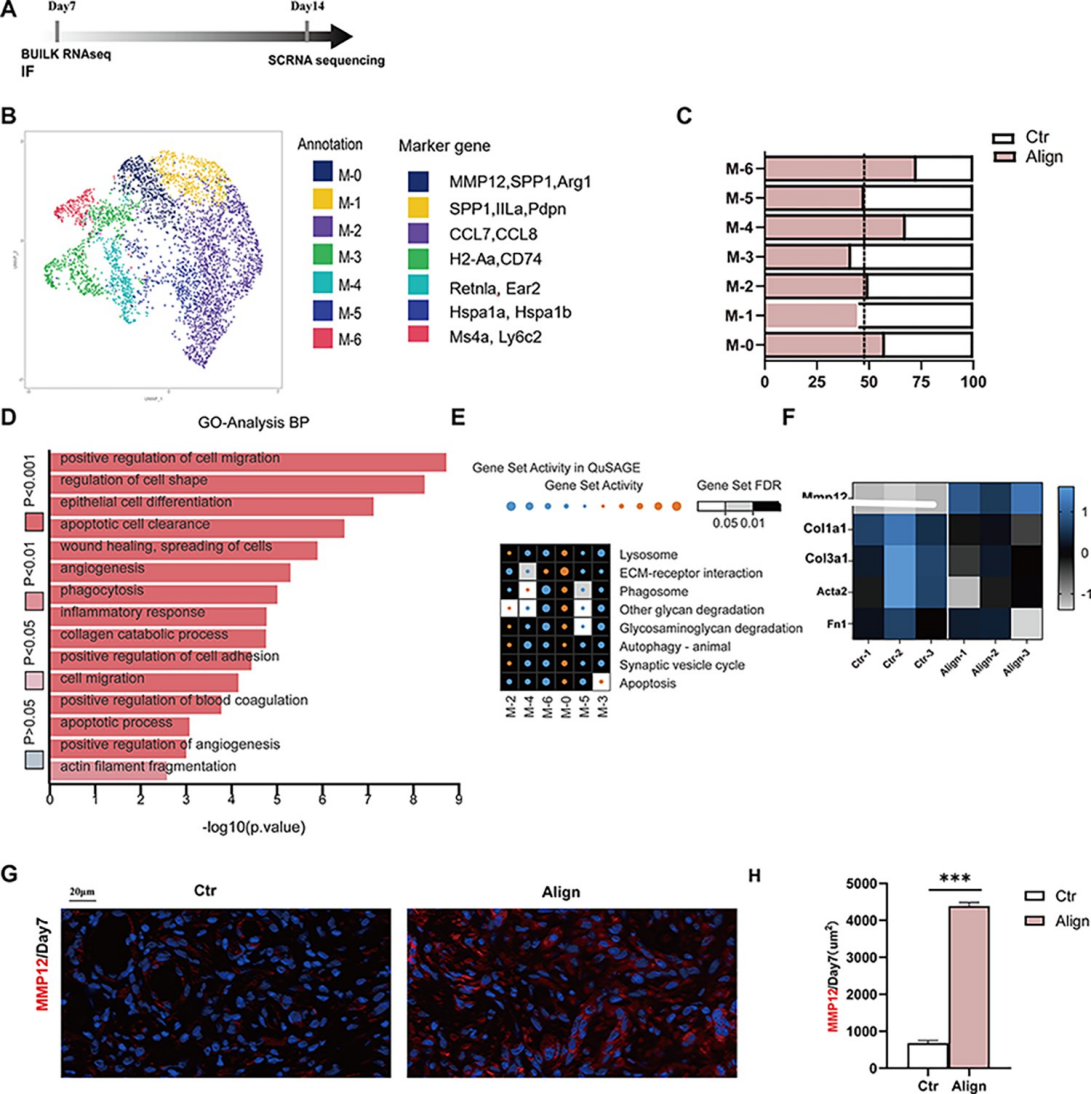
Evaluating the change of expression level of MMP12. (A) Workflow for evaluating the expression of MMP12; (B) The UMAP reduction results for the subclusters of the Mono_Mac and the annotations and marker genes are listed in the right; (C) The proportion of each subclusters of Mono_Mac; (D) The proportion and Gene Ontology (GO) enrichment results of subclusters of the macrophages in Control group and Align group; (E) The heatmap illustrating the relative activation levels of gene sets associated with subclusters in Mono_Mac; (F) The heatmap for MMP12 and genes related to scar formation in Bulk RNA sequencing; (G) MMP12 (red) positive images of IF staining of skin defects; (H) Corresponding analysis. (n = 3 for each group) Data and error bars represent the mean±s.e.m. *** P<0.001, **P <0.01, *P<0.05 and ns/no significance by Unpaired t test Student’s t-test for data in H.

The workflow of the assessment is illustrated in Fig 3A. Our previous reports have demonstrated that the capacity of macrophages to facilitate wound healing is attributable to the local regenerative immune microenvironment. Throughout all stages of skin wound healing, the dynamic and reversible phenotypic alterations of macrophages play a crucial role. Therefore, we categorize monocyte-derived macrophages in single-cell RNA sequencing (sc-RNA-seq) and further delineate their functional heterogeneity into seven subclusters (Fig 3C): M-0 (*MMP12+*, *SPP1+*, *Arg1+*), M-1 (*SPP1+*, *Arg1+*), M-2 (*CCL8+*, *CCL7+*), M-3 (*H2Aa+*, *CD74+*), M-4 *(Retnla+*, *Ear2+*), M-5 (*Hspa1a+*, *Hspa1b+*), and M-6 (*Ms4a+*, *Ly6c+*). First, we observed that MMP12 was specifically overexpressed in the M-0 subcluster. Subsequently, we analyzed this cluster to elucidate the mechanism by which MMP12 is associated with aligned membranes to promote wound healing. M-0 (*Spp1+Arg1+*) was previously considered a responder to biomaterials. It is abundant beneath the scaffold and exhibits multiple biological functions(S1H–S1K Fig) [48], it not only enhances phagocytosis to facilitate the clearance of cellular debris, but also modulates type II immune responses and participates in cell migration to promote wound healing. (Figs 3D, 3E and S1F–S1I).

**Fig 4:**
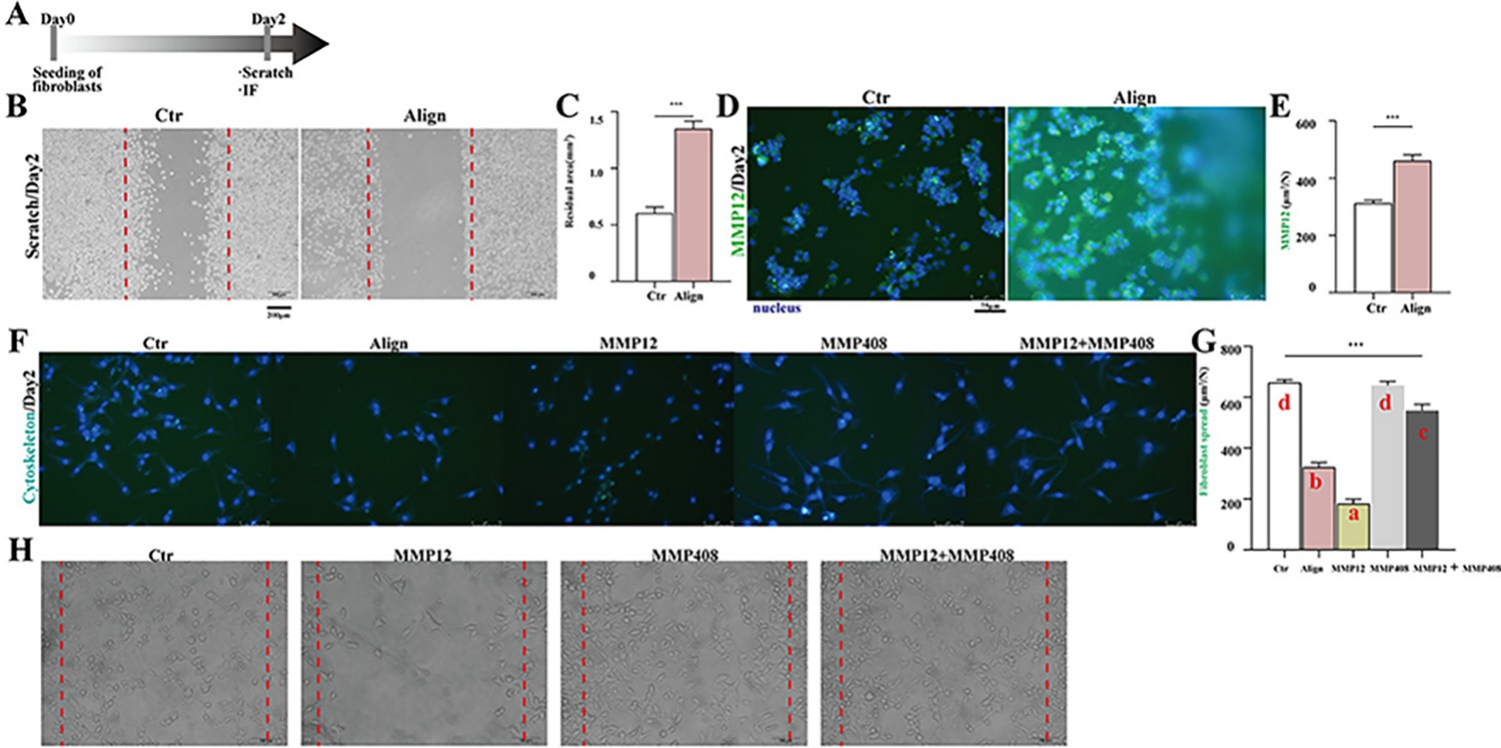
Evaluating the inhibition of MMP12 on the migration of fibroblasts. (A) Workflow for assessing the inhibitory effects of MMP12 on fibroblast migration; (B) The scratch of fibroblasts stimulated by macrophage conditioned mediums; (C) Corresponding analysis; (D) MMP12(green)-positive images of IF staining of macrophages;(E) Corresponding analysis; (F) Cytoskeleton/(green)-positive images of IF staining of fibroblasts;(G) Corresponding analysis; (H) The scratch of fibroblasts stimulated by MMP12 and/or MMP408. Different letters indicate significant differences. (n = 3 for each group) Data and error bars represent the mean±s.e.m.***P<0.001, **P<0.01, *P<0.05 and ns/no significance by Unpaired Student’s t-test for data in C and E, and ONE WAY ANOVA for data in G.

In addition, to further investigate the specific mechanisms underlying scar reduction and enhanced wound healing, we conducted Bulk RNA sequencing of skin defects in mice from both the Align group and the control group (Fig 3F) at POD 7. As illustrated in the heat map, the RNA expression levels of MMP12 were significantly elevated in the Align group compared to the Control group. Conversely, the expression of genes associated with scar formation, such as Col1a1, Col3a1, Acta2, and Fn1, was notably lower in Align than in Control. This suggests that the increased expression of MMP12 may be linked to a reduction in scar-related gene expression. Subsequently, the immunofluorescence (IF) analysis of MMP12 in the Align group (1671 ± 117.3 μm^2^) was significantly higher than that observed in the Control group (562.3 ± 39.4 μm^2^). This finding indicates that the expression of MMP12 protein is consistent with its gene expression levels (Fig 3G and 3H). Subsequently, we observed that the migration ability of fibroblasts in the Align appeared to be altered to a certain extent, as indicated by the results of Gene Ontology (GO) analysis conducted through scRNA-seq (Fig 3D). We conducted a preliminary investigation into the effects of the Align membrane and MMP12 recombinant protein on the migratory capacity of fibroblasts. As presented in Fig 4B and 4C, the residual area of scratch of fibroblasts (L929) was bigger in the aligned membrane group (macrophage conditioned medium) than in the control group (without macrophage conditioned medium), indicating that the migratory ability of fibroblasts was weaker in the Aligned group than in the control group. Accordingly, the IF of MMP12 secreted by macrophages in the aligned membrane group was much higher than in the control group (Fig 4D and 4E). The recombinant protein of MMP12 significantly diminished the spreading capacity of fibroblasts, which was partially restored by the addition of inhibitor of MMP12 (MMP408) (Fig 4F and 4G). All the results indicated that MMP12 inhibits the migratory capacity of fibroblasts. During the proliferation phase, fibroblasts and myofibroblasts within the surrounding tissues are activated to proliferate, migrate and deposit newly synthesized extracellular matrix [49]. Finally, surplus fibroblasts are removed through apoptosis, thereby preventing the excessive accumulation of extracellular matrix that can lead to scar formation [50–52].

### 3.3. MMP12 promotes wound healing

**Fig 5:**
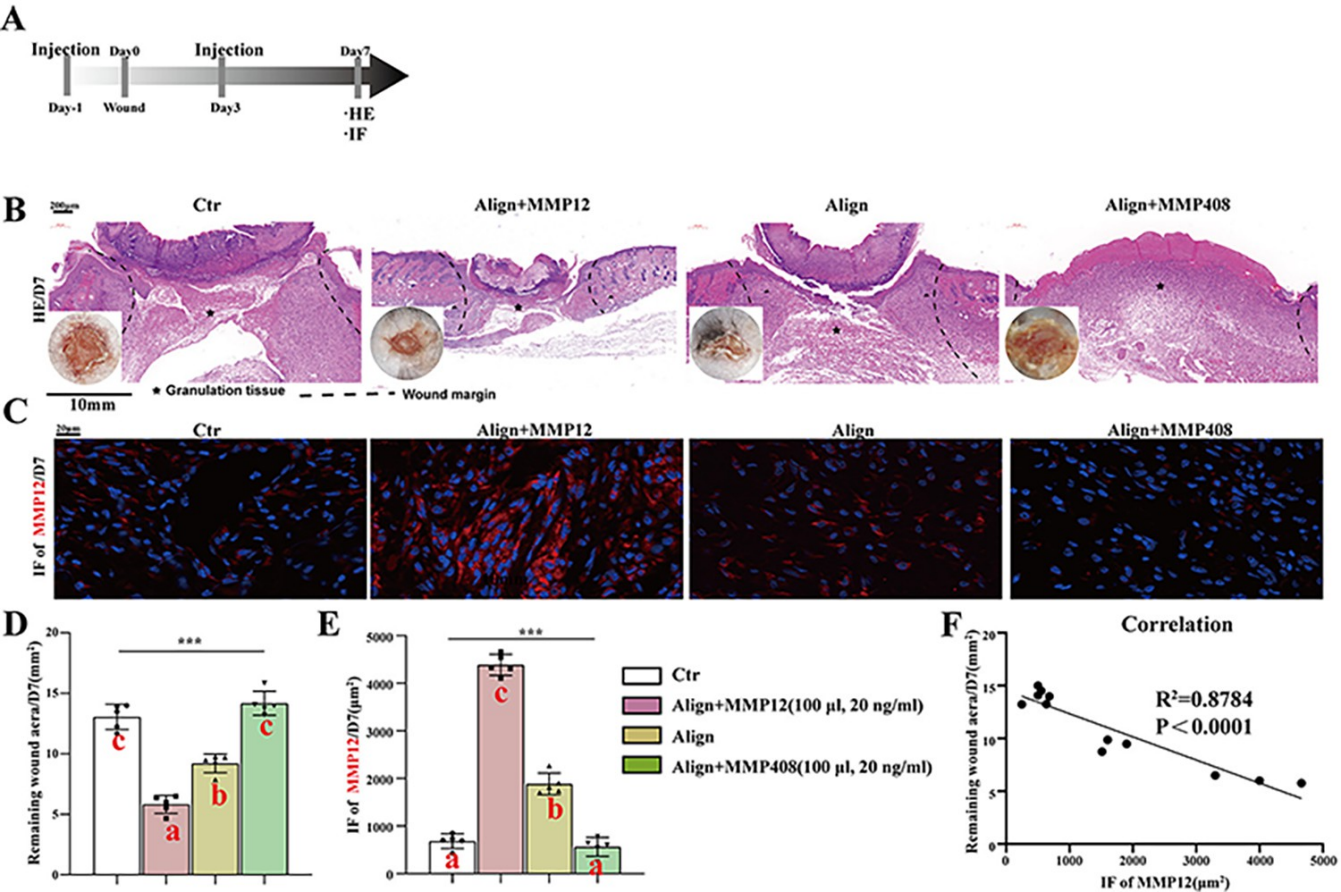
Evaluation of the effect of recombinant protein MMP12 on promoting wound healing. (A) Workflow for evaluating the inhibition of MMP12 on the wound scar formation; (B) Residual gap of H&E of skin defects stimulated by aligned membranes, MMP12 and/or MMP408; (D) Corresponding analysis; (C) MMP12(red)-positive images of IF staining of skin defects;€ Corresponding analysis; (F) The negative correlation between residual gap and MMP12. Different letters indicate significant differences. (n = 3 for each group) Data and error bars represent the mean±s.e.m. ***P<0.001, **P<0.01, *P<0.05 and ns/no significance by ONE WAY ANOVA for data in D, E and F.

The workflow for assessing the impact of MMP12 on the reduction of wound scar formation is summarized in Fig 5A. As illustrated in Fig 5B and 5D, the residual area of granulation tissue (immature scar) in mice was significantly smaller in the Align group compared to both the aligned membrane plus MMP408 group and the control group. Notably, the smallest residual area of granulation tissue was observed in the aligned membrane plus MMP12 group. The IF of MMP12 was more pronounced in Align group compared to the aligned membrane plus MMP408 and control group. Notably, the highest level of IF for MMP12 was observed in the aligned membrane plus MMP12 group. Furthermore, a significant negative correlation was observed between the expression levels of MMP12 and immature scar formation (Fig 5F). Collectively, these finding suggested that MMP12 plays an inhibitory role in the development of immature scar.

## 4. Discussion

Our study aimed to investigate the mechanisms underlying the aligned Electrospun Nanofiber Membrane that facilitates wound healing. To begin with, this study establishes that the Align membrane can enhance wound re-epithelialization, minimize scar formation, and promote hair follicle regeneration. Meanwhile, we observed a subcluster of macrophages exhibiting high expression levels of MMP12 in the scRNA-seq results. Notably, the abundance of this subcluster was significantly increased in the Align group. By analyzing this cell subcluster, it was observed that this subcluster is closely associated with cell migration, adhesion and autophagy. Adhesion and migration exhibit an inverse relationship; an optimal rate of migration is attained with increasing adhesion, yet further attachment leads to a reduction in mobility [53]. Subsequently, through Bulk RNA sequencing, we investigated the skin defects in mice from both the aligned membrane group and the control group at POD 7. Our findings revealed a significant upregulation of MMP12 RNA expression in the aligned group. Immunofluorescence experiments further confirmed that the protein expression of MMP12 secreted by macrophages was significantly higher in the Align group compared to the control group. This finding is consistent with the RNA expression results and is accompanied by a downregulation of genes associated with scar formation. This strongly supports the critical role of aligned membranes in regulating wound scar formation through MMP12. Subsequent experiments clarified the inhibitory effects of aligned membranes and recombinant MMP12 protein on fibroblast migration. In summary, we proposed that during the early stages of skin wound healing, the physical characteristics associated with the aligned membranes facilitate cell migration. Cells tend to move along the aligned membrane towards the center of the wound, thereby accelerating its closure. At POD 14, re-epithelialization is fully achieved, and the wound is conventionally regarded as healed. However, there remains a strong immune cell presence, supported by continued wound fibroblast chemokine secretion, to stimulate active fibrosis in the dermal layer [1],with the degradation of the aligned membranes. Then, the excessive expression of MMP12 inhibited the over-migration of fibroblasts to the wound and activating the process such as phagocytosis and apoptosis. This, in turn, reduced the excessive accumulation of scars [54]. In addition, Zhou and Chao et al demonstrated that MMP12 secreted by macrophages could reduce endothelial cell viability and migration by cleaving CDH5, occluding and TJP1 by establishing silicosis model [55]. The consistency of the mechanism of MMP-12-mediated migration reduction of fibroblasts and endothelial cells in both models needs further experimental confirmation. These results provide insight into mechanisms that aligned ECM scaffolds prompted wound healing and reduced scar formation and offered potential targets for future clinical treatments to enhance wound healing outcomes and reduce scar formation.

Based on this study investigating the mechanisms by which oriented electrospun fiber membranes promote wound healing in post-traumatic skin injuries, it was found that MMP12 can reduce the excessive migration of fibroblasts during the proliferation phase towards the wound site, thereby decreasing scar overproduction. In future research, these findings may lead to the development of more effective biological dressings by loading relevant factors onto oriented electrospun membranes, ultimately assisting scar patients in regaining confidence and embracing a better quality of life.

## 5. Limitations

The process of wound healing is complex, involving various cell types and molecular pathways. This study may not have encompassed all relevant factors. Furthermore, the research utilized a full-thickness skin defect model in mice (with a diameter of 6 mm), which necessitates further validation in different animal models, such as burn wound models and diabetic wound models, to broaden its applicability. Future studies are required to elucidate the underlying mechanisms more clearly and ultimately optimize strategies for addressing skin scarring step by step.

## 6. Conclusions

These findings indicate that align electrospun nanofibrous membrane can promote skin wound healing, and is closely related to the inhibition of fibroblast migration at the later stage of wound healing by high expression of MMP12.

## Supporting information

S1 Fig The expression levels of related genes and the Marker GOAnalysis in the Align group and the Ctr group of scRNA-seq results, and the relative activation degree and probability density distribution of related biological processes in M-0 subcluster. (A) The Marker GOAnalysis of HFSCs. (B) Gene expression abundance of Lgr5 of Spinous subcluster. (C) The Marker GOAnalysis of Spinous cells. (D) Krt10 gene expression abundance of Spinous cell. (E) Gene expression abundance of Krt1 of Spinous subcluster. (F) Gene expression abundance of BMP4 of Spinous subcluster. (G) Gene expression abundance of Kazald1 of Spinous subcluster. (H) The relative activation degree and probability density distribution of Apoptosis in M-0 subcluster. (I) The relative activation degree and probability density distribution of Lysosome in M-0 subcluster. (J) The relative activation degree and probability density distribution of Other glycan degradation in M-0 subcluster. (K) The relative activation degree and probability density distribution of Phagosome in M-0 subcluster.
(TIF)

S1 Raw data (XLSX)
